# Dual inhibition of CSF-1R and IDO modulates the fibrotic and immunosuppressive tumor microenvironment in pancreatic ductal adenocarcinoma

**DOI:** 10.3389/fimmu.2026.1792483

**Published:** 2026-07-15

**Authors:** Lara C. Avsharian, Suvithanandhini Loganathan, Nancy D. Ebelt, Rebecca E. Ruggiero-Ruff, Sheyla Salcido, Skye E. Inal, Meera G. Nair, Edwin R. Manuel

**Affiliations:** 1 Department of Immuno-Oncology, Beckman Research Institute of City of Hope, Duarte, CA, United States; 2 Irell and Manella Graduate School of Biological Sciences, City of Hope, Duarte, CA, United States; 3 Division of Biomedical Sciences, University of California, Riverside, Riverside, CA, United States

**Keywords:** combination therapy, drug delivery platform, immuno oncology, immunotherapy, PDAC - pancreatic ductal adenocarcinoma, tumor microenvironment

## Abstract

Pancreatic ductal adenocarcinoma (PDAC), one of the most aggressive forms of solid tumors, is characterized by extensive fibrosis and an immunosuppressive tumor microenvironment (TME) that limits therapeutic efficacy. Tumor-associated macrophages (TAMs), especially those with a pro-tumor, M2-like phenotype, contribute to immune evasion and fibrosis via TGF-β signaling and CSF-1R-mediated recruitment. Using two orthotopic PDAC models that differ in stromal composition and baseline CSF-1R expression (KPC4662.5 and Pan02), we evaluated whether therapeutic response to macrophage-targeting strategies is context dependent. Consistent with established studies, highly fibrotic KPC4662.5 tumors exhibited elevated Arg1, α-SMA, TGF-β1, and CSF-1R expression relative to Pan02 tumors. In high fibrotic, CSF1R^High^ KPC4662.5 tumors, dual targeting of CSF-1R and the immunosuppressive molecule indoleamine 2,3-dioxygenase (IDO), via PLX3397 and IDO-targeting *Salmonella*, respectively, significantly reduced tumor burden but showed no combination advantage in CSF1R^Low^ tumors. The lack of therapeutic efficacy with CSF-1R inhibitor monotherapy in PDAC models has previously been attributed to polymorphonuclear myeloid-derived suppressor cell (PMN-MDSC) infiltration following treatment. This combination therapy altered intratumoral immune cell composition by decreasing PMN-MDSCs and increasing effector T cell subsets. These findings identify tumor fibrosis and CSF-1R expression as potential biomarkers of response to combined CSF-1R and IDO inhibition and support biomarker-guided immunotherapeutic strategies in PDAC.

## Introduction

Pancreatic ductal adenocarcinoma (PDAC) is one of the most aggressive malignancies among solid tumors with a current 5-year survival rate of ~13%. It constitutes 90% of all pancreatic tumors and is projected to become the second leading cause of cancer-related death by 2030 (1). PDAC is characterized by a desmoplastic (or fibrotic) stroma which negatively affects prognosis. The fibrotic stroma, which can account for up to 90% of the tumor volume, is primarily composed of extracellular matrix (ECM) rich in hyaluronic acid and fibrillar collagen, along with activated fibroblasts, immune cells, and blood vessels. This acts as a biophysical barrier that excludes cytotoxic immune cells and promotes invasiveness (2, 3). The fibrotic tumor microenvironment (TME) acts not only as a biophysical barrier impeding drug and immune cell infiltration, but also as a biological hub that actively supports tumor progression and immune evasion.

In addition to desmoplasia, PDAC harbors an immunosuppressive TME infiltrated by tumor-associated macrophages (TAMs), myeloid-derived suppressor cell (MDSC) and regulatory T cells (Tregs) (3). Among immune infiltrates, TAMs are abundant and play a central role in PDAC progression, fibrosis and immune suppression. While classically activated (M1-like) macrophages are polarized toward a pro-inflammatory, anti-tumor phenotype, TAMs in PDAC are typically polarized toward an M2-like phenotype that promotes immune evasion, angiogenesis and tumor progression (2, 4). M2 polarized TAMs secrete immunosuppressive cytokines such as IL-10 and transforming growth factor-beta (TGF-β), facilitating tumor growth, tissue remodeling, and suppression of cytotoxic T cell activity (5).

Signaling through colony-stimulating factor 1 receptor (CSF-1R) on the surface of TAMs and TGF-β secreted by TAMs both play crucial roles in regulating fibrosis and orchestrating the recruitment and maintenance of myeloid cells within the TME. TGF-β acts as a potent pro-fibrotic cytokine by driving the differentiation of fibroblasts into activated cancer-associated fibroblasts (CAFs), which produce ECM components and contribute to both fibrosis and immunosuppression through the induction of regulatory Tregs and exclusion of effector T cells (6, 7). Simultaneously, CSF-1R signaling on the surface of TAMs promotes recruitment, survival, and M2 polarization of TAMs (8, 9). Notably, CAFs themselves secrete CSF-1, recruiting CSF-1R-positive TAMs and establishing a feed-forward loop that perpetuates fibrosis and immune suppression (10).

Given the central role of TAMs and CAFs in fostering an immunosuppressive and fibrotic TME, targeting the CSF-1/CSF-1R axis has emerged as a promising therapeutic approach. CSF-1R inhibition has been shown to deplete a subset of TAMs and partially block macrophage migration (11, 12), as well as reprogram towards an anti-inflammatory M1 phenotype, enhance anti-tumor immunity, and decrease fibrosis in solid tumors (11–14). However, the therapeutic response has been variable and limited, and recent studies suggest that the limited efficacy of CSF-1R inhibitor monotherapy in PDAC may result from compensatory recruitment of polymorphonuclear (PMN)-MDSCs following treatment. These PMN-MDSCs can functionally replace tumor-associated macrophages as dominant immunosuppressive cells within the tumor microenvironment, thereby maintaining suppression of effector T cell responses despite TAM depletion (15, 16).

Our group and others have shown that an attenuated *Salmonella typhimurium* strain engineered to carry shRNA plasmids against indoleamine 2,3-dioxygenase (shIDO-ST) activates anti-tumor functions of PMN cells in multiple solid tumor models, including PDAC. IDO is an immunoregulatory enzyme expressed abundantly in PDAC tumors that catalyzes the catabolism of tryptophan, leading to local T cell suppression and tolerance within the TME (17–20). Therefore, to circumvent therapeutic resistance to CSF-1R inhibition, we hypothesized that co-targeting CSF-1R and IDO using shIDO-ST would simultaneously deplete/reprogram TAMs and reduce PMN-MDSCs, leading to an additive or synergistic anti-tumor effect by targeting multiple immunosuppressive pathways in PDAC models.

In this study, we utilized two murine models of PDAC with distinct fibrotic profiles, Pan02 (low fibrosis) and KPC4662.5 (high fibrosis), which differ in multiple tumor microenvironmental characteristics, to assess the interplay between fibrosis and immune infiltration, particularly TAM polarization. Because these models differ markedly in stromal composition and macrophage biology, we further hypothesized that therapeutic response to CSF-1R inhibition would be context-dependent, with limited benefit in tumors exhibiting low baseline CSF-1R expression. We evaluated a combinatorial therapeutic approach using PLX3397, an FDA-approved tyrosine kinase inhibitor targeting CSF-1R (14), and shIDO-ST. Consistent with established studies in PDAC, fibrosis was associated with M2 polarization of TAMs and an immunosuppressive tumor microenvironment. Our results demonstrate that combined CSF-1R and IDO inhibition modulates the tumor immune microenvironment, reducing tumor burden in highly fibrotic, CSF-1R^high^ PDAC models.

## Material and methods

### Animals and cell lines

Transgenic 6- to 8-week-old male and female *Nramp1^r/r^* (C57BL/6 congenic strain RRID: IMSR_JAX:027081) mice were obtained from breeding colonies at City of Hope Biomedical Research Center (COH-BRC) and handled in compliance with standard IACUC guidelines. The Pan02 cell line was gifted from Dr. DC. Linehan at Washington University School of Medicine (21), while the KPC4662.5 cell line was gifted from Dr. Robert Vonderheide at the University of Pennsylvania (22). Pan02 and KPC4662.5 cells were maintained in RPMI 1640 media or DMEM media, respectively, containing 10% fetal bovine serum, 2 mM L-glutamine, 100 units/mL penicillin, and 100 μg/mL streptomycin. Prior to engraftment, cells were passaged less than five times at approximately 80% confluency.

### Orthotopic tumor engraftment

Previously published methods (23) were used for orthotopic implantation of Pan02 and KPC4662.5 cell lines into the pancreas of Nramp1 mice (n=5). To summarize, mice were anesthetized, and a small incision was made in the skin and peritoneal lining so that the pancreas could be externalized. A 27-gauge needle was used to inject 3 x 10^5^ Pan02 or KPC4662.5 cells in a volume of 50 μL of a 1:1 solution of PBS and Matrigel (Corning #354234). KPC4662.5 tumor-bearing mice were treated 14 days after engraftment and tumors were collected for immunofluorescent analysis 23 days after engraftment. In therapeutic studies with Pan02 tumor-bearing mice, treatment was initiated 6 days following surgery and mice were monitored for clinical signs of disease. Pan02-bearing mice were euthanized upon reaching humane endpoints, such as abdominal distention, lethargy and delayed response to external stimuli.

### Immunohistochemistry/immunofluorescence

Previously described methods were used (24) for both IHC and IF. Briefly, formalin-fixed paraffin-embedded (FFPE) tumor tissues were sectioned at 5 μm thickness, transferred to glass slides, deparaffinized, and rehydrated. Antigen retrieval using 10 mM Na Citrate (pH 6.0) prior to staining slides with antibodies against F4/80 (Invitrogen #MF48000, RRID: AB_10376289), Arg1 (Proteintech #16001-1-AP, RRID: AB_2289842), α-SMA (eBioscience #1409760-80, RRID: AB_2572996), TGF-β (Proteintech #21898-1-AP, RRID: AB_2811115), CSF-1R (AbboMax Inc #602-120), CD206 (R&D #AF2535, RRID: AB_2063012) and CD80 (Novus #NBP3-11976, RRID: AB_3583383). ImageJ (NIH, RRID: SCR_0166124) was used to determine collagen density in trichrome staining and to quantify brightfield IHC images. Multicolor, fluorescent images (DAPI, F4/80, CD206, and CD80) were analyzed to quantify fluorescence signal in F4/80+ cells. Quantification of other markers/targets (collagen, CSF-1R, TGF-b, and Arg1) are presented as overall raw integrated density for multiple fields (n=10). Fiji (RRID:002285) was used for channel splitting and preprocessing. F4/80+ macrophages were segmented by thresholding and particle analysis, and the resulting ROIs were applied to CD206 and CD80 channels to extract integrated density per cell, which was used to classify macrophages as CD206+ or CD80 +.

### CSF-1R inhibitor and ST administration, dosing, and therapeutic studies

Nramp1 with Pan02 or KPC4662.5 tumors were intravenously administered 2 x 10^6^ CFU shScr-ST or shIDO-ST through retro-orbital injection. CSF-1R inhibitor, PLX3397 (Tocris #7590), was reconstituted in 20% DMSO, 50% PEG300, and 30% PBS and administered simultaneously via intraperitoneal injection at a dose of 25 mg/kg. Treatments were delivered every other day for 10 days. Treatment groups were randomly assigned at the time of treatment initiation. Investigators were not blinded to treatment allocation during animal handling or downstream analyses.

### Flow cytometry

Staining of spleens and tumors was performed as previously described (25). Briefly, cells were stained with a viability dye (Invitrogen #65-0866-14) for 30 minutes at 4 °C, washed and stained with surface antibodies for 45 minutes at 4 °C. Flow cytometry antibodies used were targeted against CD45 (BD Biosciences #565478, RRID: AB_2739257, BD Biosciences #563410, RRID: AB_2738189), CD11b (BD Biosciences #553312, RRID: AB_398535), F4/80 (BD Biosciences #565411, RRID: AB_2734779), Ly6G (BD Biosciences #563005, RRID: AB_2737946), Ly6C (BD Biosciences #553104, RRID: AB_394628), CD3 (BD Biosciences #560527, RRID: AB_1737463), CD4 (BD Biosciences #560181, RRID: AB_1645235), CD8 (BD Biosciences #553033, RRID: AB_394571), PD-1 (Invitrogen #48-9985-82, RRID: AB_2574139). Flow cytometry was performed on the BD Fortessa X20 cytometer, auto-compensation was applied by BD FACSDiva software using single-stained cells, and data were analyzed using FlowJo v10.8.1 (RRID: SCR_008520) using fluorescence minus one controls.

### Statistical analysis

All statistical analyses were performed using Prism software by GraphPad (v9, RRID: SCR_002798). Data were analyzed using appropriate parametric tests (unpaired two-tailed Student’s t-tests, one-way ANOVA with Tukey’s multiple comparisons test) and non-parametric tests (Mann-Whitney tests) depending on the data distribution and experimental design. Normality assumptions were not formally tested due to limited sample size in certain experiments. Variance between groups was assessed qualitatively, and no substantial heterogeneity was observed, supporting the use of parametric tests. The two-tailed *p*-value is considered significant if *p* < 0.05. Kaplan-Meier plots were assessed using the Log-Rank test. Unless otherwise indicated, all error bars represent the standard error of the mean.

## Results

### Low-fibrotic (Pan02) and high-fibrotic (KPC4662.5) murine models of PDAC are infiltrated by macrophages.

**Figure 1:**
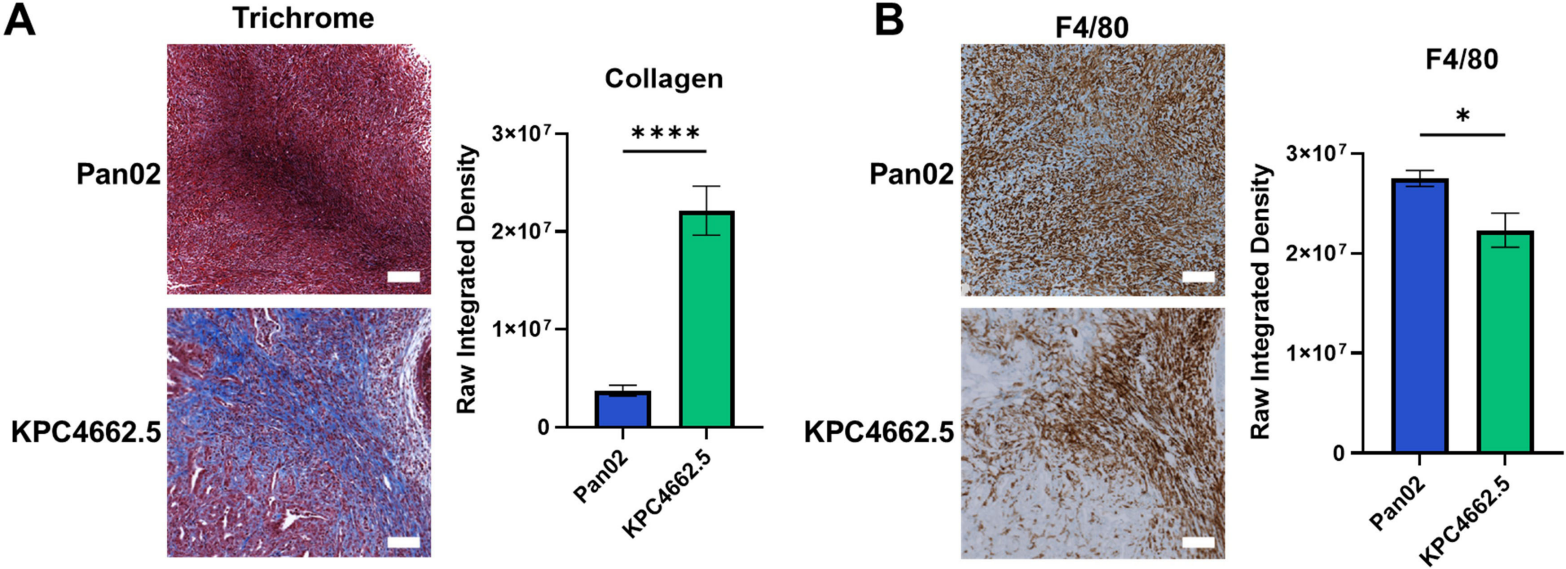
Low-fibrotic (Pan02) and high-fibrotic (KPC4662.5) murine models of PDAC are infiltrated by macrophages. **(A)** Representative images of Masson’s trichrome staining of orthotopic KPC4662.5 and Pan02 tumors established in Nramp1 mice. Collagen deposition (blue) highlights the fibrotic microenvironment in each tumor model. Quantification of collagen density confirms significantly increased fibrosis in KPC4662.5 tumors compared to Pan02 **(B)** Immunohistochemical staining for the pan-macrophage marker F4/80 in orthotopic KPC4662.5 and Pan02 tumor sections, showing macrophage infiltration. N=5. *p < 0.05, ****p < 0.0001, *t*-test. Objective: 10×. Scale bars = 100 µm.

Fibrosis is a hallmark of PDAC and blocks immune cell infiltration by acting as a biophysical barrier (2). To determine the role of TAMs in fibrosis, we compared two commonly used murine PDAC models, KPC4662.5, derived from the genetically engineered KPC mouse model shown to have dense fibrosis and an immunosuppressive microenvironment, and Pan02, a chemically induced tumor model with relatively sparse fibrosis (26). Orthotopic KPC4662.5 and Pan02 tumors were established orthotopically in Nramp 1 (*Nramp1^r/r^* C57BL/6 congenic strain RRID: IMSR_JAX:027081) mice and collagen levels were assessed using Masson’s trichrome staining (**Figure 1A**). Pan02 and KPC4662.5 tumors were both engrafted and harvested on the same day, however due to inherent differences in disease progression between tumor models, KPC4662.5 tumors were larger than Pan02 models. Consistent with previous literature (26), significantly higher collagen density was observed in KPC4662.5 tumors compared to Pan02 tumors (*p* < 0.0001, *t-test*), confirming a more fibrotic microenvironment in KPC4662.5 tumor models. We then assessed TAMs in both models using immunohistochemistry staining for a murine pan macrophage marker, F4/80 (**Figure 1B**). Both tumor models exhibited a TAM-rich microenvironment, however Pan02 tumors showed a greater density of F4/80^+^ cells. These findings are consistent with the possibility that fibrosis may limit macrophage infiltration within the tumor. However, as this comparison involves two distinct tumor models and macrophages were identified using the pan-macrophage marker F4/80, differences in macrophage density may also reflect model-intrinsic factors or differences in macrophage polarization states that are not resolved by F4/80 staining alone.

### KPC4662.5 tumors express factors that activate CAFs

**Figure 2:**
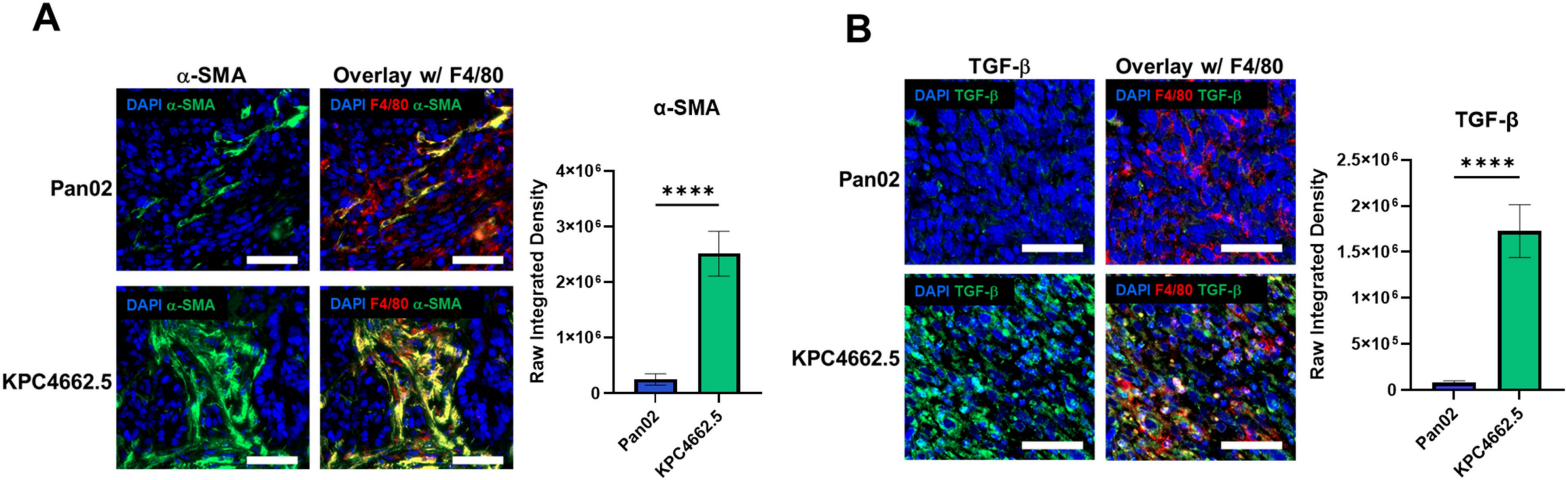
High-fibrotic (KPC4662.5) murine models express factors that signal cancer-associated fibroblasts. **(A)** Representative images of orthotopic KPC4662.5 and Pan02 tumor sections stained for α-SMA along with F4/80 and DAPI to visualize activated CAFs. Objective: 20x. Scale bars = 50 µm. **(B)** Representative images of orthotopic KPC4662.5 and Pan02 tumor sections stained for TGF-β along with F4/80 and DAPI. N=5. Objective: 40x. Scale bars = 20 µm. ****p < 0.0001, *t*-test.

To further investigate microenvironmental differences contributing to desmoplasia between Pan02 and KPC4662.5 models, we focused on CAFs. In human PDAC, CAFs are abundant and play a crucial role in tumor progression by releasing pro-tumorigenic signals that foster a more aggressive, therapy-resistant phenotype (6, 27, 28). CAFs are the primary source of ECM components such as collagen and hyaluronic acid, which create a fibrotic environment that limits immune cell infiltration and suppresses anti-tumor immunity. Activated CAFs, marked by α-SMA expression, are associated with epithelial-mesenchymal transition (EMT), poor prognosis, and enhanced tumor growth (29, 30). Therefore, we quantified the levels of α-SMA with immunofluorescent staining (**Figure 2A**). As expected, we observed significantly more α-SMA^+^ staining (*p* < 0.0001, *t-*test) in regions enriched for F4/80^+^ TAMs in KPC4662.5 tumors, which display extensive collagen deposition, compared to Pan02 tumors that exhibit comparably less collagen deposition (**Figure 1A**). CAFs in PDAC are activated by the TGF-β signaling cascade. TGF-β is a critical pro-fibrotic cytokine produced by M2-polarized TAMs that signals CAFs to increase ECM production, leads to T-cell suppression through Treg induction, and promotes EMT transition (7, 31, 32). We evaluated the levels of TGF-β in the tumor microenvironment and observed significant upregulation of TGF-β associated with F4/80^+^ TAMs in high fibrotic KPC4662.5 tumors, compared to Pan02 tumors (**Figure 2B**, *p* < 0.0001, *t*-test).These data demonstrate an association between TAM-rich fibrotic tumors, elevated TGF-β expression, and markers of CAF activation.

### Murine models of PDAC display markers of alternatively activated (M2) TAMs

**Figure 3:**
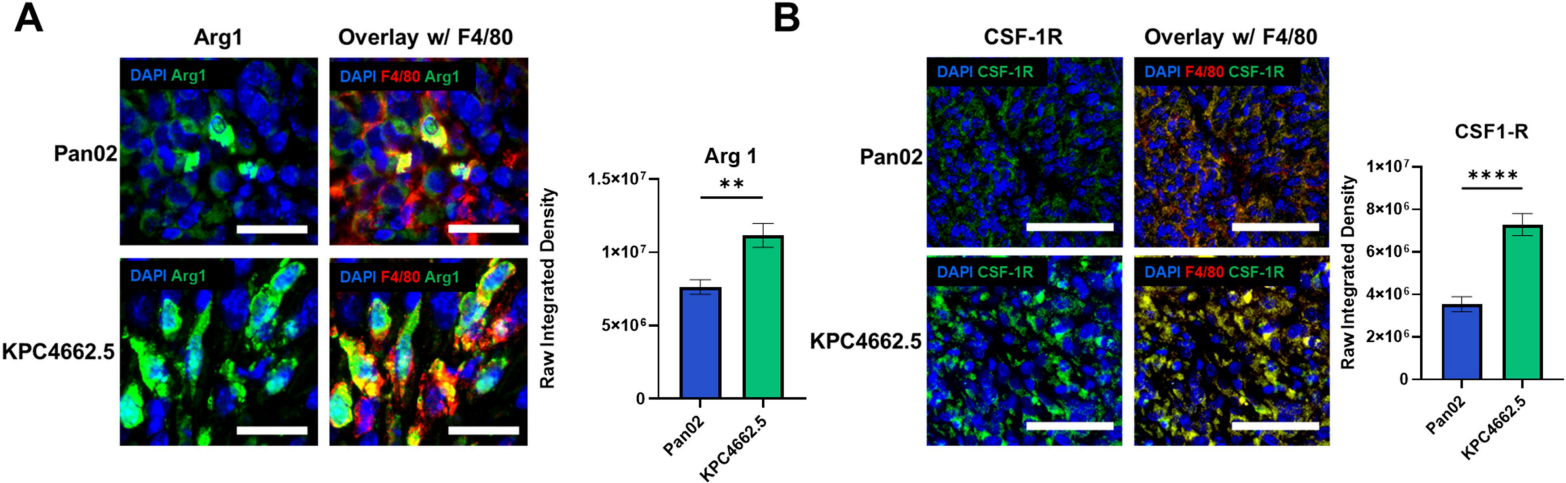
High-fibrotic (KPC4662.5) murine models of PDAC display markers of alternatively activated (M2) macrophages. **(A)** Representative immunofluorescent images of orthotopic KPC4662.5 and Pan02 tumor sections stained for Arginase-1 (Arg1), F4/80, and DAPI to visualize M2-polarized TAMs. Objective: 40×. Scale bars = 20 µm. **(B)** Representative immunofluorescent images of KPC4662.5 and Pan02 tumor sections stained for CSF-1 receptor (CSF-1R), F4/80, and DAPI. Objective: 40×. Scale bars = 20 µm. All data are expressed as mean ± standard error of the mean. N=5. **p < 0.01, ****p < 0.0001, *t*-test.

Given the elevated α-SMA and increased TGF-β levels observed in the proximity of TAMs in KPC4662.5 tumors, we hypothesized that TAMs would show significant M2 polarization in these highly fibrotic tumors. Using immunofluorescence microscopy, we assessed Arginase-1 (Arg1), a conventionally used murine M2 marker in both KPC4662.5 and Pan02 tumors to determine the polarization of TAMs (**Figure 3A**). Arg1 levels were significantly higher in KPC4662.5 tumors (*p* < 0.01, *t*-test), specifically in F4/80^+^ cells, indicating an enrichment of M2-polarized TAMs.

CSF-1 and its receptor CSF-1R have been shown to regulate several macrophage processes, including proliferation, differentiation, and survival (8). CSF-1R expression is associated with immunosuppressive TAMs, M2 polarization, and MDSCs (9). CAFs have been shown to express CSF-1 to signal the recruitment of CSF-1R^+^ macrophages into the tumor, establishing a feedback loop leading to increased CAF activation and fibrosis (10). To determine whether excess fibrosis in KPC4662.5 tumors correlates with high CSF-1R^+^ macrophage recruitment, we assessed CSF-1R expression in both tumor models overlaid with F4/80 (**Figure 3B**). As predicted, CSF-1R was significantly higher (*p* < 0.0001, *t*-test) in KPC4662.5 tumor tissues compared to Pan02. These results suggest that highly fibrotic tumors are associated with increased M2-like macrophages and elevated CSF-1R expression, features of an immunosuppressive tumor microenvironment. Having established that KPC4662.5 and Pan02 tumors differ in stromal composition and baseline CSF-1R expression, we next investigated whether these tumor features influence therapeutic response to combined CSF-1R and IDO inhibition.

### A combination of CSF-1R inhibition with shIDO-ST treatment controls orthotopic PDAC tumor growth

**Figure 4:**
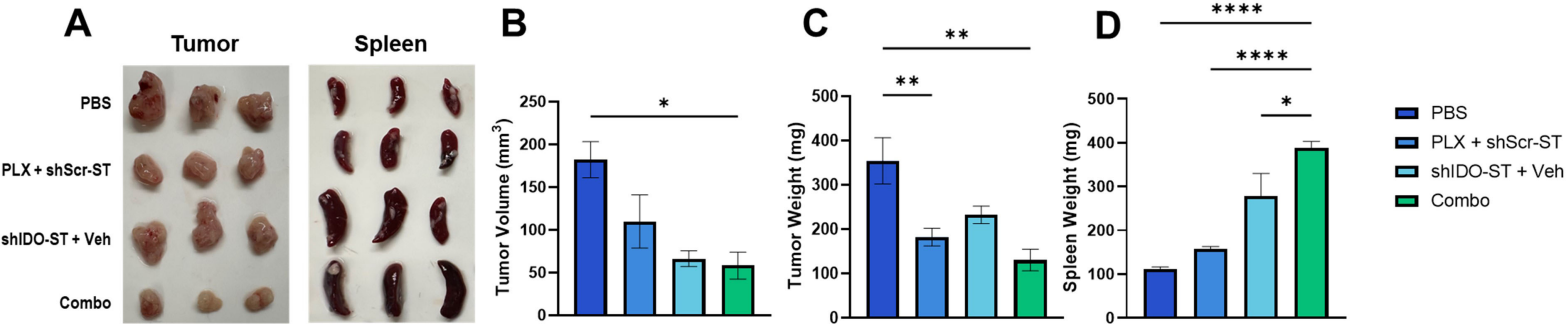
Combination of CSF-1R inhibition and shIDO-ST controls orthotopic PDAC tumor growth. Nramp1 mice bearing orthotopic KPC4662.5 tumors were treated every other day with PLX3397 (25 mg/kg), a CSF-1R inhibitor, in combination with a subtherapeutic dose of shIDO-ST (2 × 10^6 CFU) or shScr-ST controls. Twenty-four hours after the fifth dose, tumors and spleens were collected for analysis. **(A, B)** Tumor size and volume measurements from untreated (PBS), single-agent, and combination treatment groups. **(C)** Tumor weights and **(D)** spleen weights were assessed across treatment groups. Statistical analysis was performed using one-way ANOVA with Tukey multiple comparisons. N=5. *p < 0.05, **p < 0.01, ****p < 0.0001.

In multiple tumor models, including PDAC, CSF-1/CSF-1R inhibition has failed to control tumor growth, and CSF-1R inhibition led to an increase in PMN-MDSCs (15, 16). Our group has previously shown that solid tumor models treated with shIDO-ST leads to an infiltration of activated, anti-tumor PMNs. In these studies, shIDO-ST has been previously shown to reduce IDO expression and enzymatic activity across multiple tumor models (17–20). Therefore, we hypothesized that activating PMNs using shIDO-ST may improve the therapeutic efficacy of CSF-1R inhibition by preventing the accumulation or immune suppressive functions of PMN-MDSCs, ultimately resulting in enhanced tumor growth suppression. To determine the therapeutic response of this combination, we utilized PLX3397 (PLX), an FDA-approved tyrosine kinase inhibitor targeting CSF-1R, alone or in combination with a subtherapeutic dose of shIDO-ST. Mice bearing orthotopic KPC4662.5 tumors were treated every other day with 25 mg/kg PLX or vehicle (Veh) and 2 x 10^6^ CFU shIDO-ST or shScramble (shScr)-ST controls. In this design, comparison between shIDO-ST and shScr-ST groups enables distinction between IDO-specific effects and those attributable to the Salmonella vector. Twenty-four hours after the 5^th^ dose, when there was a palpable difference in tumor size between the untreated (PBS) and treated groups. Mice were euthanized, and tumor and spleen tissues were collected for *ex vivo* analysis. We opted to assess therapeutic efficacy at experimental endpoints, and refrain from survival studies in KPC4662.5 tumor-bearing mice due to technical limitations in accurately determining when a humane endpoint was reached in this tumor model. KPC-luciferase-expressing cells that allow for *in vivo* monitoring of orthotopic pancreatic tumor growth are highly immunogenic (33), which may skew therapeutic outcomes, and manual palpation is approximate, allowing for tumor overgrowth without any visible signs of morbidity. In KPC4662.5 tumor-bearing mice, where we observed high CSF-1R expression (**Figure 3B**), there was a significant decrease in tumor size and volume (*p* < 0.01, ANOVA) in mice treated with the combination compared to the untreated treatment group (**Figures 4A, B**). Although statistically insignificant, we do also observe a decrease in tumor size with the single-agents, suggesting that the combination treatment may allow for an additive therapeutic effect. We also observed a significant decrease in tumor weight compared to the PBS group in the PLX single-agent group and the combo group (*p* < 0.001, ANOVA) (**Figure 4C**) and a significant increase in spleen weight in the combination group compared to both the untreated and single-agent groups (*p* < 0.01, 0.001, ANOVA) (**Figure 4D**) which is expected with systemic *Salmonella* infection and suggests systemic immune alterations or increased myeloid output as a response to treatment (34, 35). These results indicate that CSF-1R inhibition, in combination with shIDO-ST, can inhibit tumor growth in CSF-1R^high^ tumors over the course of treatment.

To determine if this combination is effective in CSF-1R^low^ models, we tested the combination treatment in mice bearing orthotopic Pan02 tumors. The Pan02 cell line was established through chemical induction, which initiated multi-organ tumors, and Pan02 cells were expanded from pancreatic tumors (26). This may explain why the orthotopic Pan02 model, unlike the orthotopic KPC4662.5 model, does not form solid, palpable tumors and instead presents with lethal metastases and ascites within 21 days of engraftment. For these reasons, we chose to conduct survival analyses with Pan02 engrafted mice. Mice were administered the same dosing regimen as KPC4662.5 tumor-bearing mice. After 5 treatments, we assessed survival probability (**Supplementary Figure 1**). We began treating Pan02 tumor-bearing mice, a CSF-1R^low^ model, six days post-engraftment, and mice were euthanized when visible signs of disease were observed (i.e., abdominal distension, lethargy, and delayed response to external stimuli). Autopsies were performed to observe the extent of metastases with each treatment (**Supplementary Figure 2**). We observed that treatment with PLX3397 increased survival in Pan02 tumor-bearing mice compared to untreated groups (*p* < 0.01, Log-rank), and a combination with shIDO-ST expanded survival significantly more. We also observed increased survival in the shIDO-ST only group compared to the untreated control (*p* < 0.01, Log-rank). No difference in survival between the shIDO-ST and the combination group was observed (*p* > 0.5293, Log-rank). These results suggest that in KPC4662.5, a CSF-1R^high^ model, CSF-1R inhibition in combination with shIDO-ST treatment leads to a possible additive or enhanced therapeutic response. However, in Pan02, a CSF-1R^low^ model, shIDO-ST monotherapy is sufficient to extend survival, and an enhanced therapeutic response was not observed when combined with CSF-1R inhibition.

### CSF-1R inhibition depletes intra-tumoral M2-polarized macrophages in KPC4662.5 tumors

**Figure 5:**
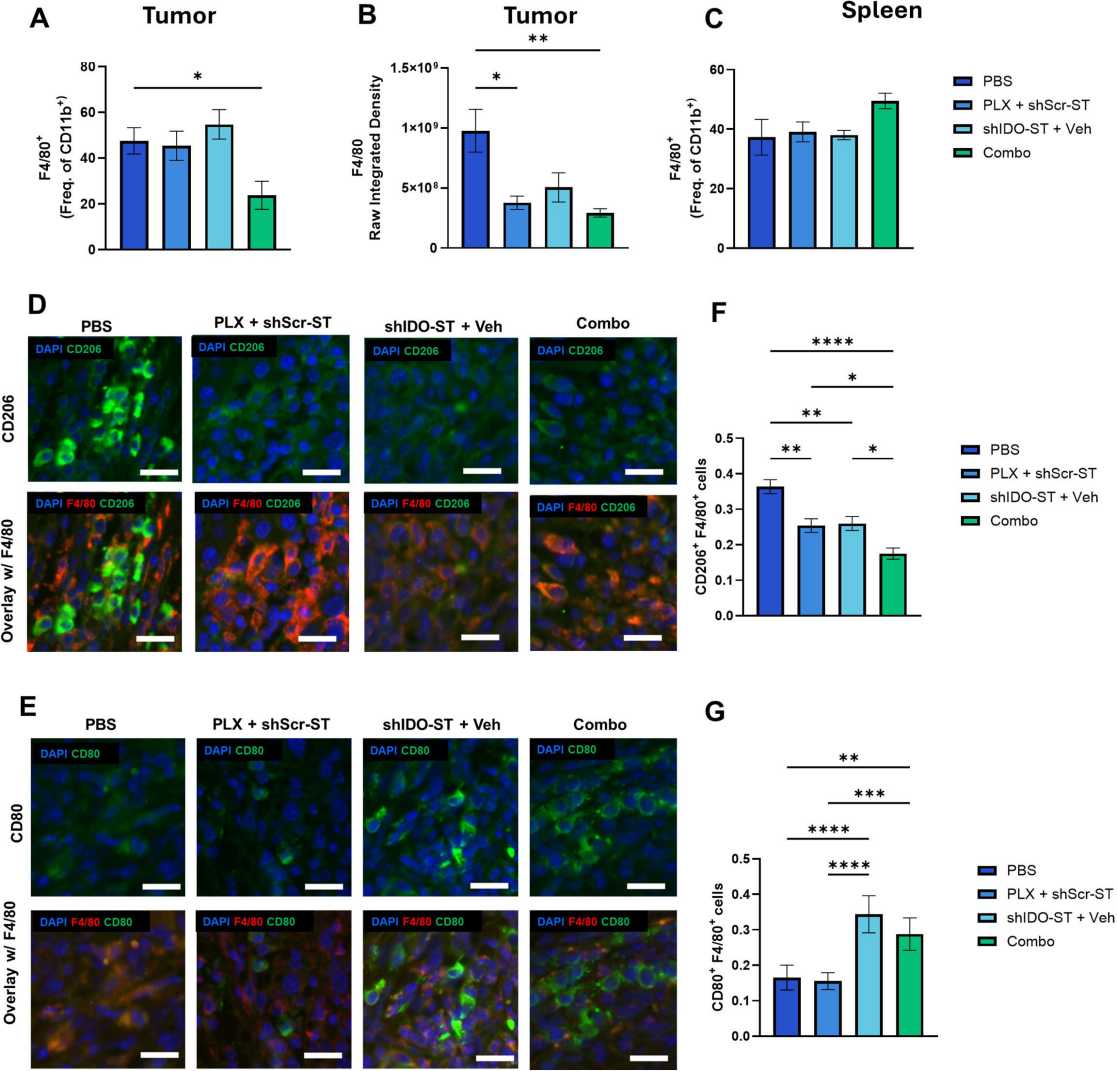
Combination treatment with CSF-1R inhibitor and shIDO-ST reduces intratumoral macrophages and M2 phenotype. **(A)** Flow cytometric quantification of F4/80^+^ macrophages in KPC4662.5 tumors. *t*-tests were used for statistical analysis. N=4 **(B)** Immunohistochemical quantification of F4/80^+^ macrophages in tumor sections. One way ANOVA was used for statistical analysis. N=4. **(C)** Flow cytometric analysis of F4/80^+^ macrophages in spleens from all treatment groups. N=4. **(D)** Representative immunofluorescent images of KPC4662.5 tumor sections stained for CD206, F4/80, and DAPI. **(E)** Representative immunofluorescent images of KPC4662.5 tumor sections stained for CD80, F4/80, and DAPI. **(F)** Fraction of CD206^+^ F4/80^+^ cells among total F4/80^+^ cells. One way ANOVA was used for statistical analysis. N=5. **(G)** Fraction of CD80^+^ F4/80^+^ cells among total F4/80^+^ cells. N=5. One way ANOVA was used for statistical analysis. *p < 0.05, **p < 0.01, ***p < 0.001, ****p < 0.0001. Objective: 20X. Scale bars = 20 µm.

To elucidate immunomodulatory changes that contribute to therapeutic effects of CSF-1R inhibitor and shIDO-ST combination treatment, we assessed immune cell populations within the treated tumors. The mechanism of PLX3397 is well characterized in multiple solid tumor types in that CSF-1R inhibition decreases intratumoral macrophages (11, 12). Therefore, we assessed TAM (F4/80^+^) quantity and density in excised KPC4662.5 tumors by flow cytometry (**Figure 5A**) and immunohistochemistry (**Figure 5B**). Significant depletion of TAM by flow cytometry was observed between the combination treatment group compared to the untreated group (*p* < 0.01, *t-*test). We observed a statistically significant decrease in F4/80^+^ cells in the tumor in the CSF-1R only group compared to the untreated group by immunofluorescent microscopy (**Figure 5B**), but not by flow cytometry (**Figure 5A**). This difference may reflect localized effects in response to CSF-1R inhibition alone, as immunofluorescence allows assessment of spatially preserved and biologically relevant niches of the tumor. Flow cytometry, on the other hand, due to whole-tumor digestion for analysis, captures the overall heterogeneity of the tumor, including areas with necrosis and hypoxia, providing a broader analysis of the tumor microenvironment. Furthermore, statistically significant decreases were not observed in the shIDO-ST group compared to untreated control, suggesting that IDO inhibition does not contribute to overall macrophage depletion.

With immunohistochemistry, a significant decrease in macrophage density was observed between the untreated group and the PLX + shScr-ST group (*p* < 0.01, ANOVA), with a more pronounced reduction in the combination treatment group (*p* < 0.001, ANOVA). These results indicate that PLX3397 depletes TAM in CSF-1R^high^ tumors. F4/80 levels remained unchanged in the spleens of mice from all treatment groups (**Figure 5C**), demonstrating that PLX3397 at 25mg/kg per mouse, every other day, affects TAM levels but does not affect splenic macrophage levels, suggesting that the effects of CSF-1R inhibition are localized to the tumor microenvironment rather than systemic. CSF-1R inhibitors have also been shown to reprogram macrophages from an M2 phenotype to an M1 phenotype (36). Therefore, we assessed TAM polarization by immunofluorescent staining for CD206, a well-established M2 marker (**Figure 5D**), and CD80, a commonly used M1 marker (**Figure 5E**). Quantification of CD206^+^ F4/80^+^ cells (i.e., M2-like TAMs) revealed a significant reduction in M2-like TAMs in tumors treated with PLX3397 (*p* < 0.01, ANOVA), indicating that CSF-1R inhibition decreases M2-like TAMs in PDAC compared to untreated control. Interestingly, shIDO-ST monotherapy also led to a modest but significant decrease in M2-like TAMs (*p* < 0.01, ANOVA), supporting prior findings that IDO inhibition may promote macrophage repolarization toward an inflammatory phenotype (37). Notably, the combination treatment group (PLX3397 + shIDO-ST) exhibited a highly significant reduction in CD206^+^ F4/80^+^ cells compared to untreated tumors and single agent-treated tumors (**Figure 5F**, p < 0.0001, ANOVA), indicating a potentially synergistic effect on reducing M2 TAMs. We next assessed M1 macrophage levels by quantifying the fraction of CD80^+^ F4/80^+^ cells (i.e., M1-like TAMs). A significant increase in M1-like macrophage fraction was observed in both the shIDO-ST (*p* < 0.0001, ANOVA) and combination (**Figure 5G**, *p* < 0.01, ANOVA) treatment groups compared to untreated controls. These findings suggest that IDO inhibition primarily promotes an inflammatory, M1-like phenotype in TAMs, while CSF-1R blockade depletes M2-like TAMs, collectively leading to an anti-tumor response. Consistent with the defined mechanism of action of PLX3397, these results convey that CSF-1R inhibition results in a depletion of TAMs, specifically immunosuppressive, pro-tumoral M2 TAMs. In contrast, IDO inhibition influences macrophage polarization, shifting the balance of TAMs in the TME from an M2 phenotype to a more inflammatory, anti-tumoral M1.

### A combination of CSF-1R inhibition and shIDO-ST is associated with decreased suppressive and increased effector immune subsets

**Figure 6:**
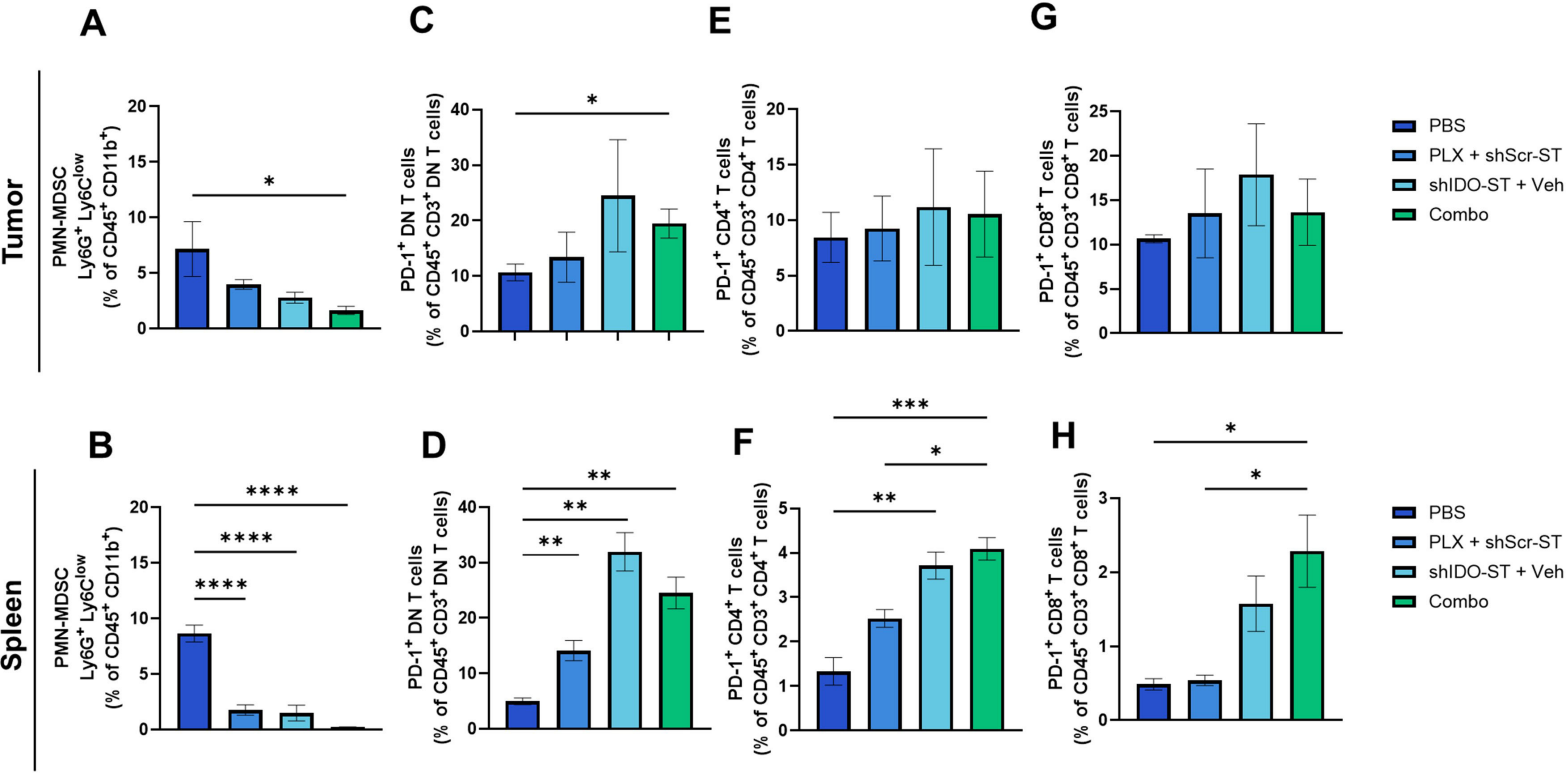
A decrease in M2 macrophages is associated with a decrease in immunosuppressive immune subsets and an increase in effector cell subsets in tumor and spleen. **(A, B)** Flow cytometry analysis of polymorphonuclear myeloid-derived suppressor cells (PMN-MDSCs; CD11b^+^ Ly6G^+^ Ly6C^low^) in tumor and spleen samples from orthotopic KPC4662.5 tumor-bearing mice across treatment groups. **(C-H)** Quantification of activated T cell subsets, including PD-1^+^ double negative (DN) T cells **(C, D)**, CD4^+^**(E, F)**, and CD8^+^ **(G, H)** T cells in tumor **(C, E, G)** and spleen **(D, F, H)** samples from treated and untreated mice. All data are expressed as mean ± standard error of the mean. Statistical analysis was performed using one-way ANOVA and *t*-tests. N=5. *p < 0.05, **p < 0.01, ***p < 0.001, ****p < 0.0001.

As previously stated, we hypothesized that shIDO-ST could mitigate the influx of PMN-MDSC that limits therapeutic efficacy of CSF-1R inhibition. To test this, we analyzed PMN-MDSC populations in both tumors and spleens across all treatment groups using flow cytometry (**Figures 6A, B**). In the tumor, we observed a significant decrease in the intratumoral percentage of myeloid cells that express PMN-MDSC markers (CD11b^+^ Ly6G^+^ Ly6C^low^) (38) in the combination treatment group compared to the untreated control (*p* < 0.01, ANOVA). However, we do not see a significant difference in the PMN MDSC counts in single-agent treated tumors in comparison to the untreated tumors, suggesting that CSF-1R inhibition and IDO inhibition in combination are necessary for PMN-MDSC decrease. In the spleen, PMN-MDSC levels were reduced across all treatment groups relative to the untreated group (*p* < 0.0001, ANOVA). These results imply that there may be an initial effect on PMN phenotype within the spleen, and a combination of CSF-1R and IDO inhibition allows that effect to be carried out in the tumor, as only the combination group had significantly decreased PMN-MDSCs in the tumor. We did not observe changes in overall myeloid cell levels in either tumor or spleen (**Supplementary Figures 3A, B**), but we did see a change in the myeloid populations, with a decrease in PMNs and an increase in monocytes within the tumor and spleen with single-agent treatments and combination treatment (**Supplementary Figures 3C–F**), suggesting that both agents influence myeloid phenotype. We also assessed T cell populations within the tumor and spleen. There was no difference observed in CD4^+^ T cells across all groups in both spleens and tumors (**Supplementary Figures 4A, B**). An increase in CD8^+^ T cells in the spleen was observed with CSF-1R monotherapy (**Supplementary Figure 4C**), which is consistent with previous studies that have found that CSF-1R inhibition results in an increased CD8^+^/CD4^+^ T cell ratio (39). A decrease in CD8^+^ T cells in the spleen was observed between the untreated group compared to the combination group, however no change was seen between the untreated and shIDO-ST only group. We observed an increase in double-negative (DN) T cells in the spleen with the combination treatment, but no changes in single-agent groups (**Supplementary Figure 4E**). Further investigation is necessary to accurately interpret these results, however these data might reflect T cell plasticity within the spleen. No significant changes in T cell numbers were observed in the tumors of all groups (**Supplementary Figures 4D, F**), suggesting a lack of migration to the tumor. We then specifically assessed PD-1^+^ T cell populations and observed that, in tumor, elevated PD-1^+^ DN (CD4^-^CD8^-^) T cells were present in the combination group compared to untreated controls (*p* < 0.01, *t-*test) (**Figure 6C**), with no change between the untreated group and the single-agent groups, implying that the combination of CSF-1R inhibition and shIDO inhibition is necessary for infiltration of activated DN T cells into the tumor. The level of activated CD4^+^ and CD8^+^ T cells in tumors across all treatment groups remained unchanged (**Figures 6E, G**). In the spleen, activated DN T cells were elevated across all treatment groups (*p* < 0.001, *t*-test) (**Figure 6D**), indicating that CSF-1R inhibition alone and shIDO-ST alone contribute to the systemic activation of DN T cells, displaying a joint effect in the combination treatment. Interestingly, DN T cells have previously been shown to act as effector cells in PDAC models, restricting migration and proliferation of tumor cells (40). There was also an increase in activated CD4^+^ (**Figure 6F**) and CD8^+^ (**Figure 6H**) T cells in the spleens of the combination group compared to untreated controls and the PLX + shScr-ST group (*p* < 0.01, ANOVA). The shIDO-ST group compared to the untreated group showed increased PD-1^+^CD4^+^ T cells, while there was no change between the PLX and untreated group, implying that increased activation of CD4^+^ T cells seen in the combination treatment group is an IDO-mediated effect. There was no significant difference in the percentage of splenic PD-1^+^CD8^+^ T cells in the combination group compared to the shIDO-ST group, and between the shIDO-ST group and the untreated group. These data lead us to believe that the increase in splenic PD-1^+^CD8^+^ T cells observed with the combination treatment may be modestly attributable to IDO inhibition with further enhancement when combined with CSF-1R inhibition. Overall, these data indicate that the combination treatment causes an activation of T cell populations within the spleen and suggests that DN T cells play an important role in PDAC progression. Further studies on their effective function in PDAC are essential to developing immunotherapeutic treatments for PDAC.

## Discussion

In PDAC, CSF-1R expression is associated with a poor prognosis, and targeting the CSF-1/CSF-1R axis is believed to be a promising strategy for depleting or reprogramming immunosuppressive TAMs and fibrosis (41). Although the pro-tumoral and pro-fibrotic roles of CSF-1R-positive macrophages have been extensively documented in PDAC, whether tumor fibrosis and baseline CSF-1R expression influence therapeutic response to macrophage-targeted therapies remains incompletely understood. Our findings demonstrate that combined CSF-1R and IDO inhibition selectively benefits highly fibrotic, CSF-1R^high^ PDAC models, suggesting that stromal context may influence treatment efficacy and patient selection. Consistent with previous studies of CSF-1R inhibition in pancreatic tumor models (13, 15), we show that PLX3397 reduces overall TAM density and immunosuppressive TAMs within the TME of murine PDAC models, yet the therapeutic effect is modest and limited. CSF-1R inhibition leads to increased CXCL1 expression from CAFs and in turn, increased infiltration of PMN-MDSCs in both spleens and tumors of multiple syngeneic cancer lines, including PDAC, through signaling of the CXCR2^+^ receptor (13, 16). In this manner, with CSF-1R inhibition only, the tumor microenvironment appears to substitute the immunosuppressive action of TAMs with the T cell suppressive actions of PMN-MDSCs.

PMN-MDSCs are instrumental in modulating the T-cell response in normal physiology, however, in cancer, PMN-MDSCs suppress antigen-specific T-cell proliferation in a dose-dependent manner (38). IDO expressed from tumor cells is associated with MDSC infiltration and inhibition of IDO has significantly limited MDSC infiltration in solid tumors (42). Furthermore, MDSCs have been shown to produce IDO, resulting in Treg stimulation and expansion (43). For this reason, we chose to use a combinatorial approach to treat PDAC, combining PLX3397 with a subtherapeutic dose of shIDO-ST to simultaneously deplete TAMs and mitigate infiltrating PMN-MDSCs to achieve tumor growth control. Indeed, this combination resulted in a significant reduction in tumor volume in CSF-1R^high^ PDAC murine models. We did not see an increase in intratumoral PMN-MDSC frequency with PLX3397 monotherapy, as Kumar et al. (16) reported in Lewis lung carcinoma (LLC) tumor-bearing mice treated with JNJ-40346527, another small molecule CSF-1R inhibitor, or Zhu et al. (15) reported in Kras-INK (KI) tumor-bearing mice treated with PLX3397. However, Candido et al. (13) reported no changes in MDSC frequency following treatment with a highly specific CSF-1R inhibitor, AZD7507, in KPC models. PLX3397, although primarily a CSF-1R inhibitor, also inhibits FMS-like tyrosine kinase 3 (FLT3) and c-Kit (8). This leads us to hypothesize that changes in MDSC frequency following CSF-1R inhibition are context dependent, varying based on the tumor model characteristics. We did however observe a decrease in PMN-MDSC levels in the tumor following combination treatment compared to no treatment controls, suggesting that both TAM depletion and IDO inhibition are necessary for PMN-MDSC mitigation in KPC4662.5 tumor models. To further iterate the significance of varying tumor models on the effects of therapeutic interventions, a modest survival advantage was observed in a CSF-1R^low^ PDAC murine model (Pan02) following PLX3397 administration, that did not result in an additive therapeutic effect when combined with shIDO-ST, as it did in the CSF-1R^high^ KPC4662.5 model. This highlights the importance of evaluating biomarkers to predict therapeutic response and selecting appropriate and representative tumor models for developing immunomodulatory agents.

PLX3397 in combination with shIDO-ST treatment appears to achieve tumor growth control in PDAC models. We hypothesize that this is a result of an enhanced T cell response. In tumors treated with PLX3397 and shIDO-ST, we saw a systemic increase in PD-1^+^ T cells and an increase in the PD-1^+^ DN T cells within the tumor. Although PD-1 is an inhibitory receptor on T cells, PD-1 expression is upregulated following stimulation of the T cell receptor, making PD-1 a marker of T cell activation. In various solid cancers, including PDAC, infiltration of activated PD-1^+^ lymphocytes is associated with better clinical outcomes and longer overall survival (44–46). DN T cells have been shown to stop PDAC tumor cell expansion and migration through the Fas/FasL pathway in both *in vitro* culture conditions and *in vivo* using a PANC-1 xenograft model treated with DN T cells (40, 47). Moreover, PD-1^+^ DN T cells represented a subset of T cells that are self-reactive and pro-inflammatory, while PD-1^-^ DN T cells represented an anti-inflammatory phenotype (48). Our data associates a decrease in tumor burden with an increase in PD-1^+^ intratumoral DN T cells, suggesting a self-reactive and pro-inflammatory role for PD-1^+^ T cells in KPC4662.5 tumors following treatment with PLX3397 and shIDO-ST. However, it is important to note that PD-1 is an inhibitory receptor commonly used as a marker of T-cell exhaustion, and therefore functional analyses of T-cell activity are necessary to determine whether PD-1^+^ DN T cells have an activated phenotype and contribute to anti-tumor immunity. Furthermore, additional studies are required to confirm the mechanisms of DN T cell priming and migration following PLX3397 and shIDO-ST combination treatment. One potential source of double negative T cells is CD8^+^/CD4^+^ T cells that downregulate coreceptor expression upon exposure to cognate self-antigens in the periphery (49–51). In our study, we observed increased splenic PD-1^+^ CD8^+^, CD4^+^, and DN T cells. In our previous studies with shIDO-ST treatment in PDAC, we saw an activation of anti-tumor neutrophils within the spleen, that then migrated to the tumor, where they exhibited cytotoxic effects (17). We hypothesize that similarly, the spleen may serve as the site of DN T cell priming, potentially through interactions with activated CD8^+^ and CD4^+^ T cells, that results in tumor infiltration by DN T cells. Nonetheless, our results suggest an influential role for DN T cells in PDAC treatment, and further research to elucidate their specific functions is essential for understanding the PDAC tumor microenvironment and enhancing immunotherapeutic interventions.

At first glance, the observation that highly fibrotic KPC4662.5 tumors exhibit lower overall macrophage density compared to Pan02 tumors may appear inconsistent with the subsequent finding that macrophage numbers decrease further following combination therapy. However, these findings reflect different comparisons. Baseline immunofluorescence analyses compare untreated tumors across models and indicate that dense fibrosis limits overall macrophage infiltration in KPC4662.5 tumors. Importantly, the macrophages that do infiltrate these tumors are enriched for CSF-1R expression and an M2-like phenotype. In contrast, therapeutic studies assess changes within the same tumor model, demonstrating that CSF-1R inhibition further depletes this suppressive macrophage population rather than contradicting the initial baseline observation. Additionally, a limitation of the baseline immunofluorescence analyses is the potential confounding effect of tumor burden, as Pan02 tumors were smaller than KPC tumors at harvest, despite identical engraftment-to-harvest timelines.

We note that combinatorial targeting of CSF-1R and IDO has been explored previously in other tumor contexts, demonstrating that concurrent disruption of macrophage and tryptophan mediated immunosuppression can enhance anti-tumor immunity. However, the present study differs in several important aspects. First, we explicitly stratify therapeutic response by tumor fibrosis and baseline CSF-1R expression using two orthotopic PDAC models, revealing a context- dependent benefit of CSF-1R inhibition that is restricted to highly fibrotic, CSF-1R^high^ tumors. Second, IDO targeting in this study is achieved through a tumor-colonizing Salmonella-based shRNA delivery platform rather than small-molecule inhibition, enabling localized immune modulation and distinct myeloid reprogramming effects. The use of shIDO-ST specifically allows for activation of innate immunity, both through recognition of pathogen-associated molecular patterns and IDO silencing, whereas the objective of small molecule inhibitors of IDO is to activate adaptive anti-tumor immunity. In our previous studies characterizing shIDO-ST in PDAC, we saw that myeloid cell activation toward an anti-tumor phenotype was associated with phagocytosis of ST by PMNs both in spleens and tumors, leading to the recruitment of cytotoxic PMNs (17, 18, 20). In this manner, robust targeting of PMN-MDSC infiltration following CSF-1R inhibition is achieved by shIDO-ST. Third, our findings identify double-negative T cells as a potentially relevant effector population following combination therapy. Together, these features distinguish the current work from prior combination studies and provide new mechanistic insight into how stromal context and myeloid composition influence response to macrophage- and IDO-targeted therapies in PDAC. While our findings are supported by consistent trends across multiple experimental readouts, the relatively small number of biological replicates in certain *in vivo* experiments may limit the statistical power to detect more subtle effects. Increasing cohort sizes and independent replication of these studies will be important to further validate the robustness and reproducibility of the observed therapeutic responses. Additionally, several analyses were conducted with relatively small sample sizes, which limited formal assessment of normality assumptions. Accordingly, both parametric and non-parametric statistical approaches were employed where appropriate, however, larger cohort sizes in future studies will further strengthen statistical robustness and reproducibility. While analyses were conducted using standardized quantification approaches, investigators were not blinded to treatment allocation, which may introduce potential bias in more subjective assessments such as tumor palpation and immunohistochemical evaluation. Future studies incorporating blinded analyses will further strengthen these as observations. In conclusion, this study demonstrates that CSF-1R expression and macrophage phenotype are associated with fibrosis and immune suppression in PDAC, and that combined targeting of CSF-1R and IDO has potential as an immunotherapeutic intervention, particularly in highly fibrotic, CSF-1R^high^ tumors.

While our findings reveal associations between fibrosis, macrophage phenotype, and therapeutic response, additional mechanistic studies will be required to establish causal relationships between CSF-1R^+^ macrophages, CAF activation, and fibrosis development in PDAC.

## Data Availability

The original contributions presented in the study are included in the article/**Supplementary Material**. Further inquiries can be directed to the corresponding author.

## Supplementary material

Supplementary Figure 1 Survival analysis of Pan02-bearing mice treated with CSF-1R inhibitor and shIDO-ST combination therapy. Nramp1mice bearing orthotopic Pan02 tumors were treated with PLX3397 (25 mg/kg), in combination with shIDO-ST (2 × 10^6 CFU) or shScr-ST controls. Treatments were initiated 6 days post-tumor engraftment and administered every other day for five doses. Survival probability was monitored until humane endpoints were reached due to metastatic disease progression. Kaplan-Meier survival curves depict differences in survival across treatment groups. Statistical analysis was performed using the pairwise log-rank tests. N=4. ** *p* < 0.01.

Supplementary Figure 2 Significant metastases and ascites observed in Pan02 model regardless of treatment. Autopsies were performed on Nramp1 mice bearing orthotopic Pan02 tumors after five doses of PLX3397 (25 mg/kg), shIDO-ST (2 × 10^6^ CFU), combination therapy, or untreated (PBS) controls. Representative images show the extent of metastatic spread and ascites formation across treatment groups. Increased metastatic burden and ascites were observed in untreated mice **(A)**, while treated groups exhibited variable reductions in metastatic disease **(B-D)**. White arrows show primary tumors; red arrows point metastasis.

Supplementary Figure 3 Changes in myeloid cell populations following CSF-1R inhibitor and shIDO-ST combination treatment. **(A, B)** Flow cytometry analysis of myeloid cells (CD11b^+^) in spleen and tumor samples from orthotopic KPC4662.5 tumor-bearing mice across treatment groups. **(C, D)** Flow cytometry analysis of PMNs (Ly6G^+^) in CD4^+^ T cells in spleen and tumor samples from orthotopic KPC4662.5 tumor-bearing mice across treatment groups. **(E, F)** Flow cytometry analysis of monocytes (F4/80^-^ Ly6G^-^ Ly6C^+^) in tumor and spleen samples from orthotopic KPC4662.5 tumor-bearing mice across treatment groups. All data are expressed as mean ± standard error of the mean. Statistical analysis was performed using one way ANOVA. N=5. * *p* < 0.05, ** *p* < 0.01, **** *p* < 0.0001.

Supplementary Figure 4 Changes in lymphoid cell populations following CSF-1R inhibitor and shIDO-ST combination treatment. **(A, B)** Flow cytometry analysis of CD4^+^ T cells in spleen and tumor samples from orthotopic KPC4662.5 tumor-bearing mice across treatment groups. **(C, D)** Flow cytometry analysis of CD8^+^ T cells in spleen and tumor samples from orthotopic KPC4662.5 tumor-bearing mice across treatment groups. **(E, F)** Flow cytometry analysis of DN T cells in spleen and tumor samples from orthotopic KPC4662.5 tumor-bearing mice across treatment groups. All data are expressed as mean ± standard error of the mean. Statistical analysis was performed using one-way ANOVA. N=5. * *p* < 0.05, ** *p* < 0.01, *** *p* < 0.001.

Supplementary Figure 5 Representative tumor sample for myeloid cell gating. Gating was done on FMO samples. Major cell populations (F4/80^-^Ly6C^+^Ly6G^-^ Monocytes, F4/80^+^ Macrophages, Ly6G^+^ PMNs, and Ly6G^+^Ly6C^low^ PMN-MDSCs) are indicated in red.

Supplementary Figure 6 Representative spleen sample for myeloid cell gating. Gating was done on FMO samples. Major cell populations (F4/80^-^Ly6C^+^Ly6G^-^ Monocytes, F4/80^+^ Macrophages, Ly6G^+^ PMNs, and Ly6G^+^Ly6C^low^ PMN-MDSCs) are indicated in red.

Supplementary Figure 7 Representative spleen sample for lymphoid cell gating. Gating was done on FMO samples. Major cell populations (CD3^+^CD4^+^ T cells, CD3^+^CD8^+^ T cells and CD3^+^CD4^-^CD8^-^ DN T cells) are indicated in red.

Supplementary Figure 8 Representative tumor sample for lymphoid cell gating. Gating was done on FMO samples. Major cell populations (CD3^+^CD4^+^ T cells, CD3^+^CD8^+^ T cells and CD3^+^CD4^-^CD8^-^ DN T cells) are indicated in red.
