# Supplementary figures and images for: Dual inhibition of CSF-1R and IDO modulates the fibrotic and immunosuppressive tumor microenvironment in pancreatic ductal adenocarcinoma
Source: Front Immunol. 2026 Jul 15;17:1792483. doi: 10.3389/fimmu.2026.1792483 (PMC13416583; doi:10.3389/fimmu.2026.1792483)

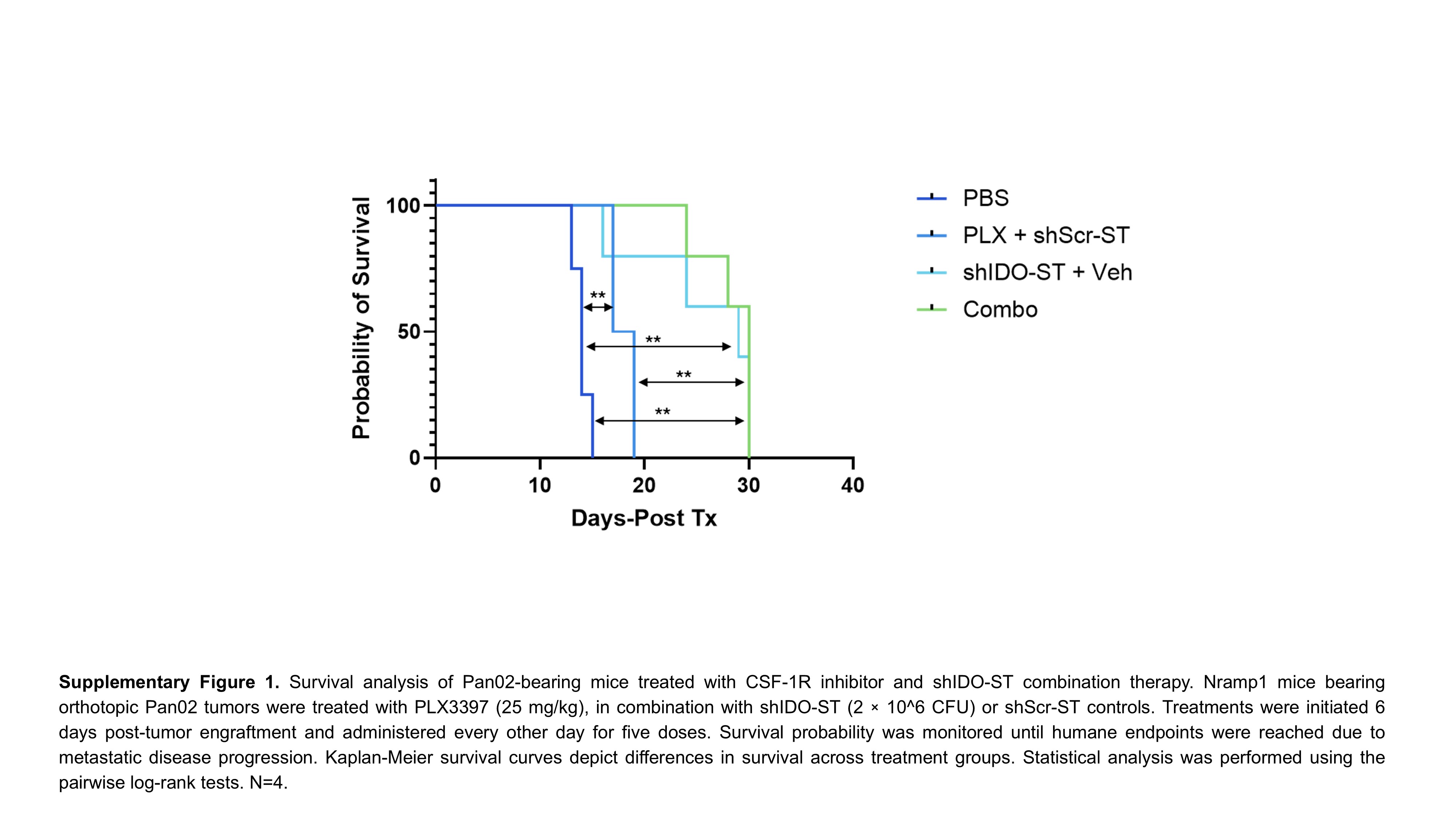

Supplement: Supplementary Figure 1 — Survival analysis of Pan02-bearing mice treated with CSF-1R inhibitor and shIDO-ST combination therapy. Nramp1mice bearing orthotopic Pan02 tumors were treated with PLX3397 (25 mg/kg), in combination with shIDO-ST (2 × 10^6 CFU) or shScr-ST controls. Treatments were initiated 6 days post-tumor engraftment and administered every other day for five doses. Survival probability was monitored until humane endpoints were reached due to metastatic disease progression. Kaplan-Meier survival curves depict differences in survival across treatment groups. Statistical analysis was performed using the pairwise log-rank tests. N=4. ** p < 0.01. [file Image1.jpeg]

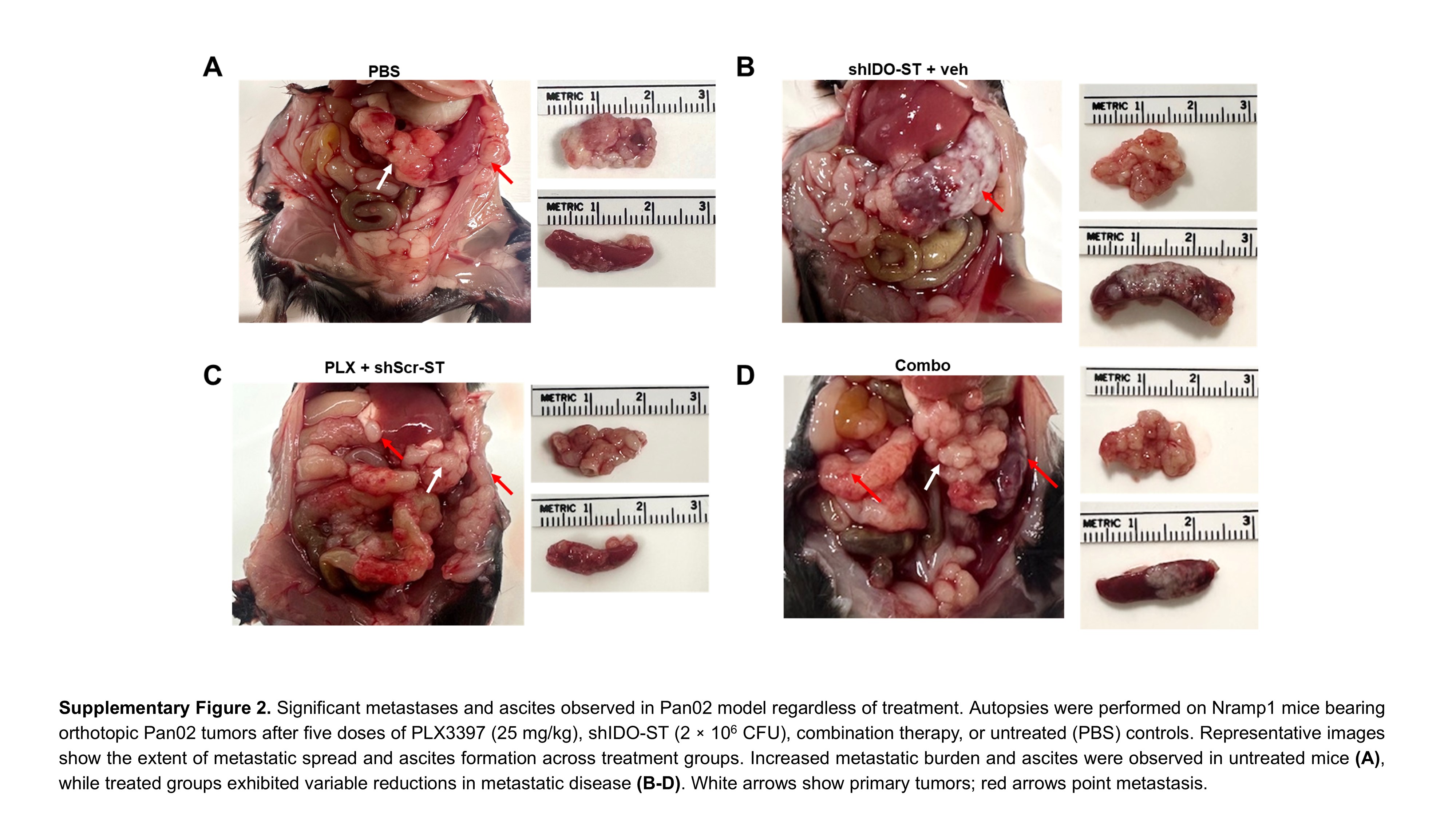

Supplement: Supplementary Figure 2 — Significant metastases and ascites observed in Pan02 model regardless of treatment. Autopsies were performed on Nramp1 mice bearing orthotopic Pan02 tumors after five doses of PLX3397 (25 mg/kg), shIDO-ST (2 × 10^6^ CFU), combination therapy, or untreated (PBS) controls. Representative images show the extent of metastatic spread and ascites formation across treatment groups. Increased metastatic burden and ascites were observed in untreated mice (A), while treated groups exhibited variable reductions in metastatic disease (B-D). White arrows show primary tumors; red arrows point metastasis. [file Image2.jpeg]

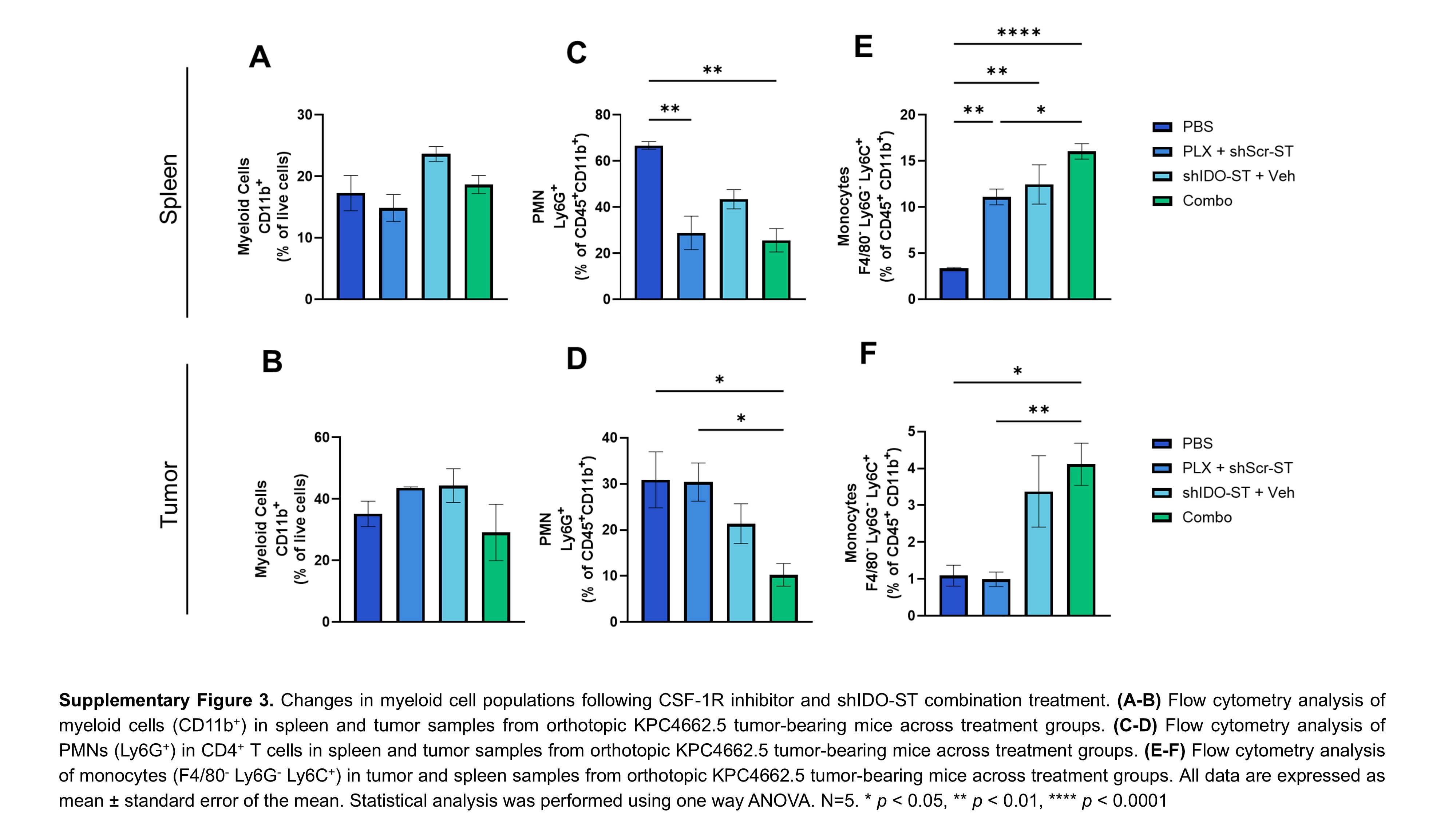

Supplement: Supplementary Figure 3 — Changes in myeloid cell populations following CSF-1R inhibitor and shIDO-ST combination treatment. (A, B) Flow cytometry analysis of myeloid cells (CD11b+) in spleen and tumor samples from orthotopic KPC4662.5 tumor-bearing mice across treatment groups. (C, D) Flow cytometry analysis of PMNs (Ly6G+) in CD4+ T cells in spleen and tumor samples from orthotopic KPC4662.5 tumor-bearing mice across treatment groups. (E, F) Flow cytometry analysis of monocytes (F4/80- Ly6G- Ly6C+) in tumor and spleen samples from orthotopic KPC4662.5 tumor-bearing mice across treatment groups. All data are expressed as mean ± standard error of the mean. Statistical analysis was performed using one way ANOVA. N=5. * p < 0.05, ** p < 0.01, **** p < 0.0001. [file Image3.jpeg]

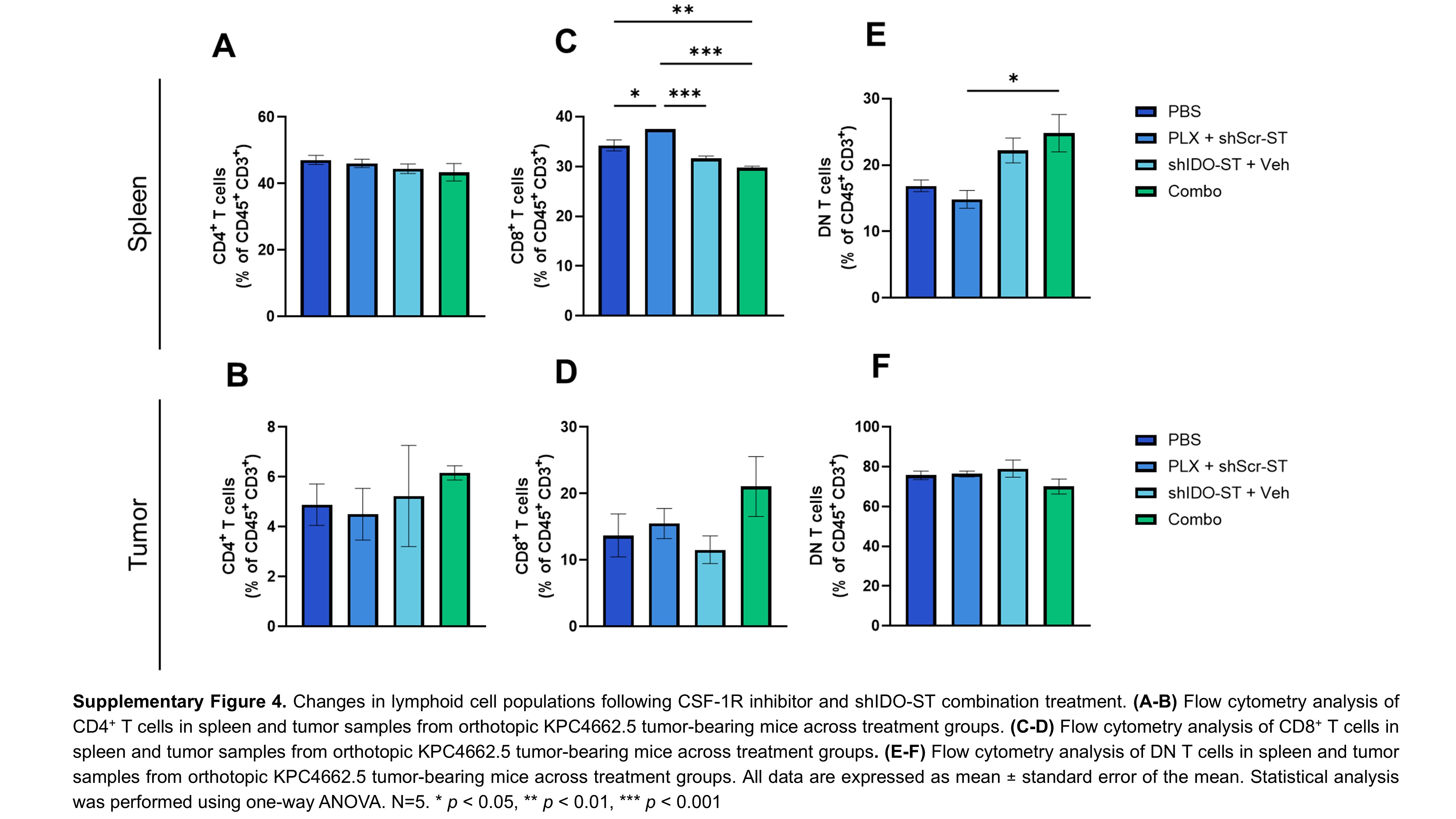

Supplement: Supplementary Figure 4 — Changes in lymphoid cell populations following CSF-1R inhibitor and shIDO-ST combination treatment. (A, B) Flow cytometry analysis of CD4+ T cells in spleen and tumor samples from orthotopic KPC4662.5 tumor-bearing mice across treatment groups. (C, D) Flow cytometry analysis of CD8+ T cells in spleen and tumor samples from orthotopic KPC4662.5 tumor-bearing mice across treatment groups. (E, F) Flow cytometry analysis of DN T cells in spleen and tumor samples from orthotopic KPC4662.5 tumor-bearing mice across treatment groups. All data are expressed as mean ± standard error of the mean. Statistical analysis was performed using one-way ANOVA. N=5. * p < 0.05, ** p < 0.01, *** p < 0.001. [file Image4.jpeg]

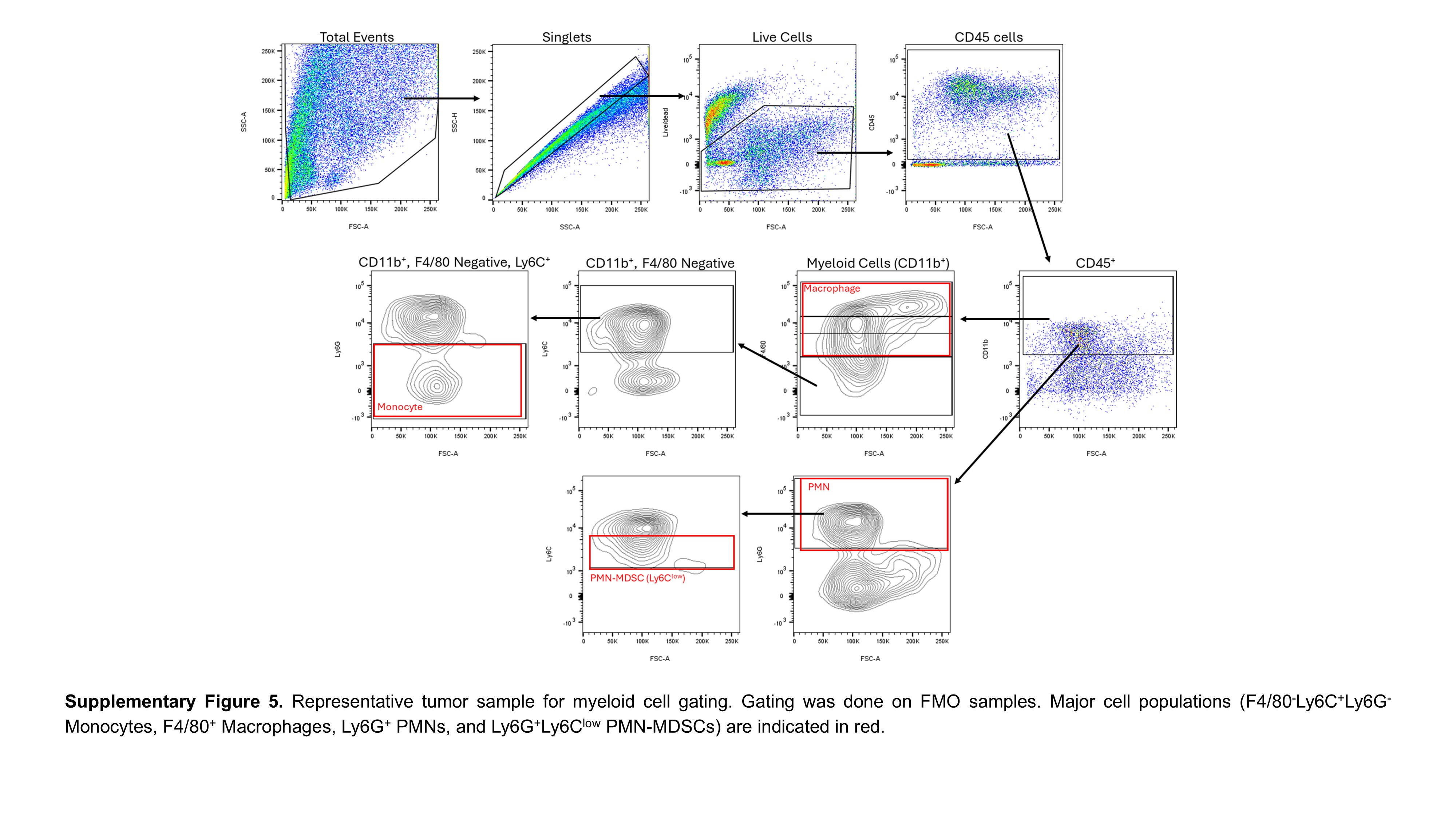

Supplement: Supplementary Figure 5 — Representative tumor sample for myeloid cell gating. Gating was done on FMO samples. Major cell populations (F4/80-Ly6C+Ly6G- Monocytes, F4/80+ Macrophages, Ly6G+ PMNs, and Ly6G+Ly6Clow PMN-MDSCs) are indicated in red. [file Image5.jpeg]

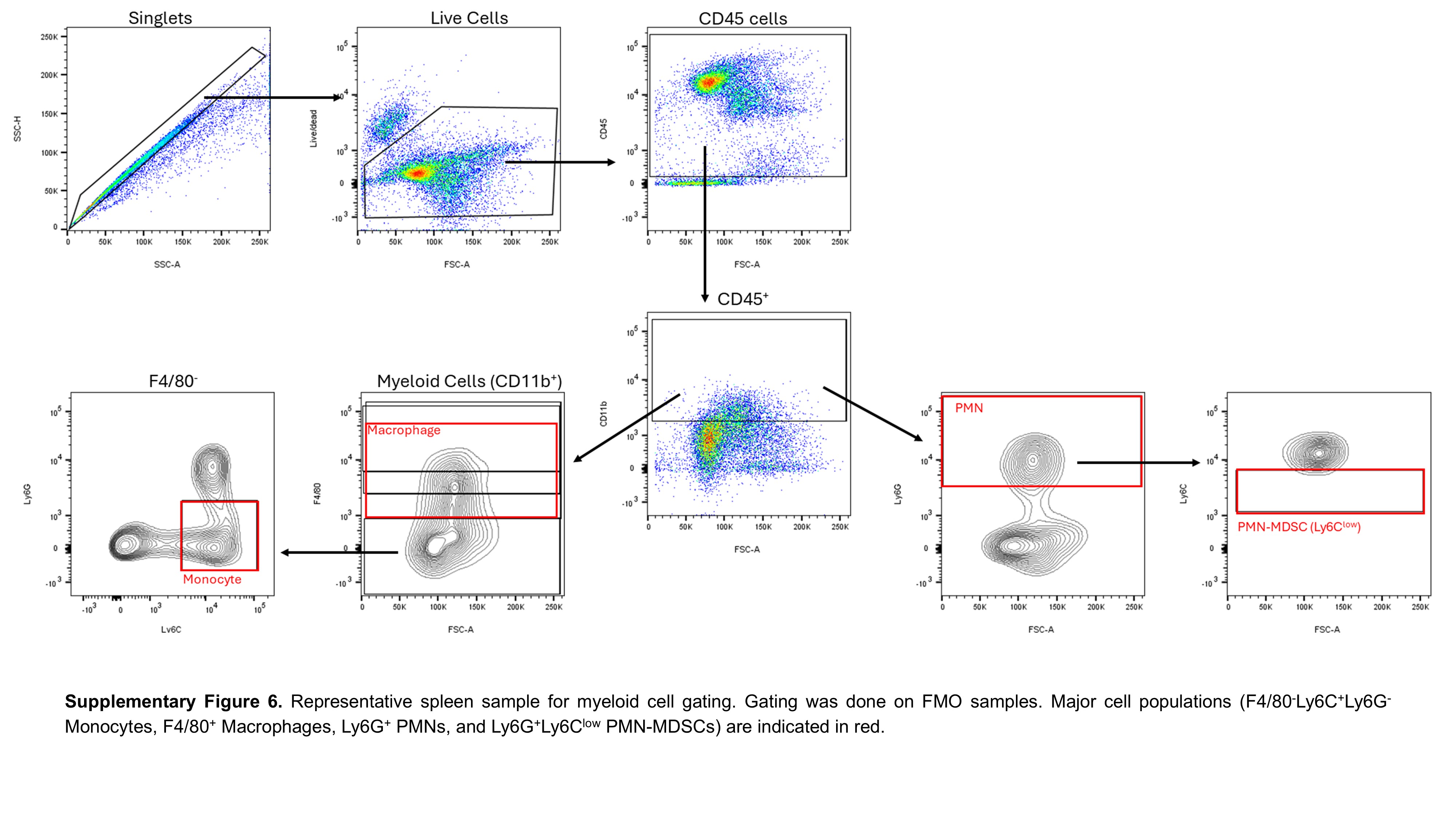

Supplement: Supplementary Figure 6 — Representative spleen sample for myeloid cell gating. Gating was done on FMO samples. Major cell populations (F4/80-Ly6C+Ly6G- Monocytes, F4/80+ Macrophages, Ly6G+ PMNs, and Ly6G+Ly6Clow PMN-MDSCs) are indicated in red. [file Image6.jpeg]

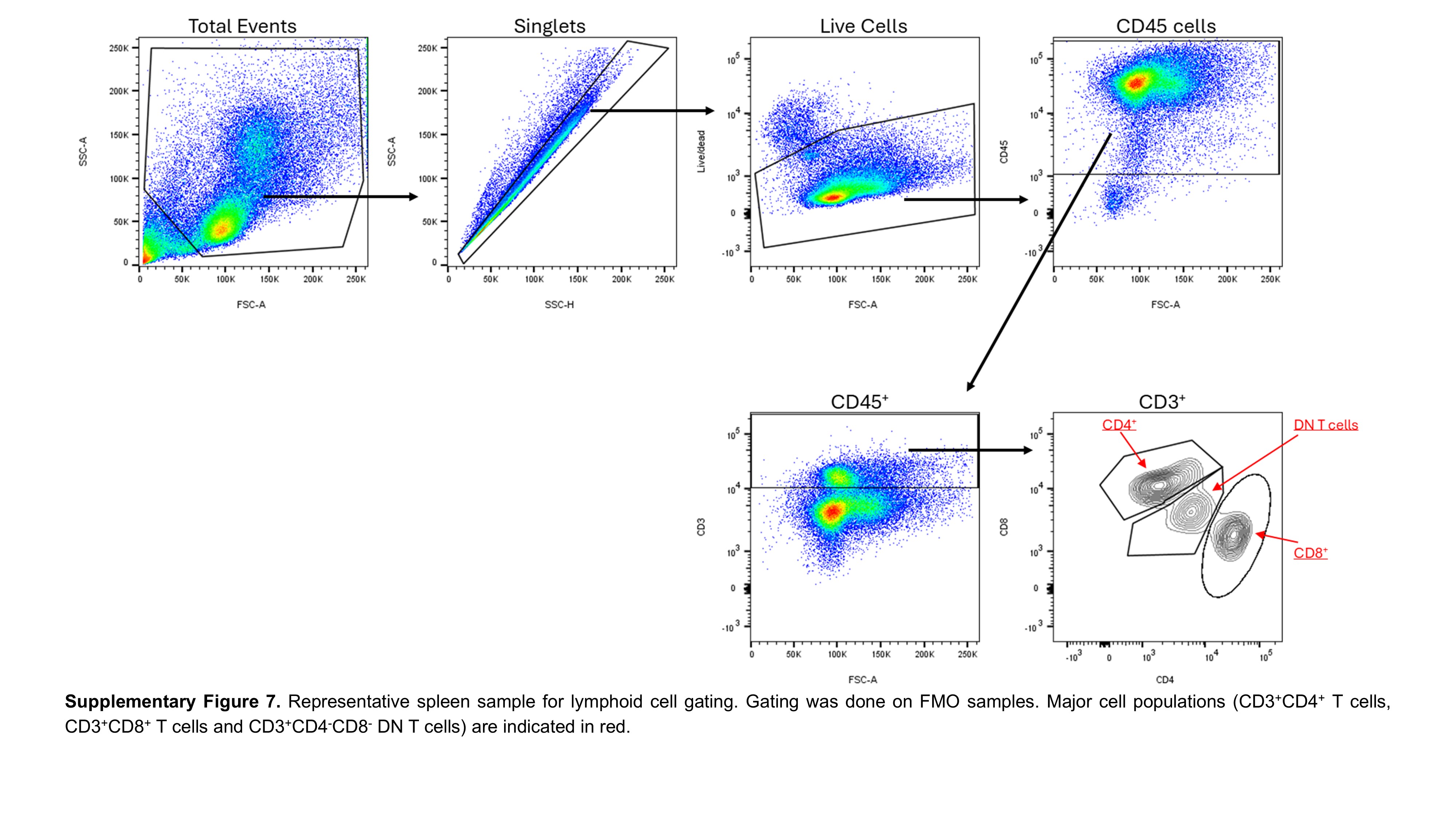

Supplement: Supplementary Figure 7 — Representative spleen sample for lymphoid cell gating. Gating was done on FMO samples. Major cell populations (CD3+CD4+ T cells, CD3+CD8+ T cells and CD3+CD4-CD8- DN T cells) are indicated in red. [file Image7.jpeg]

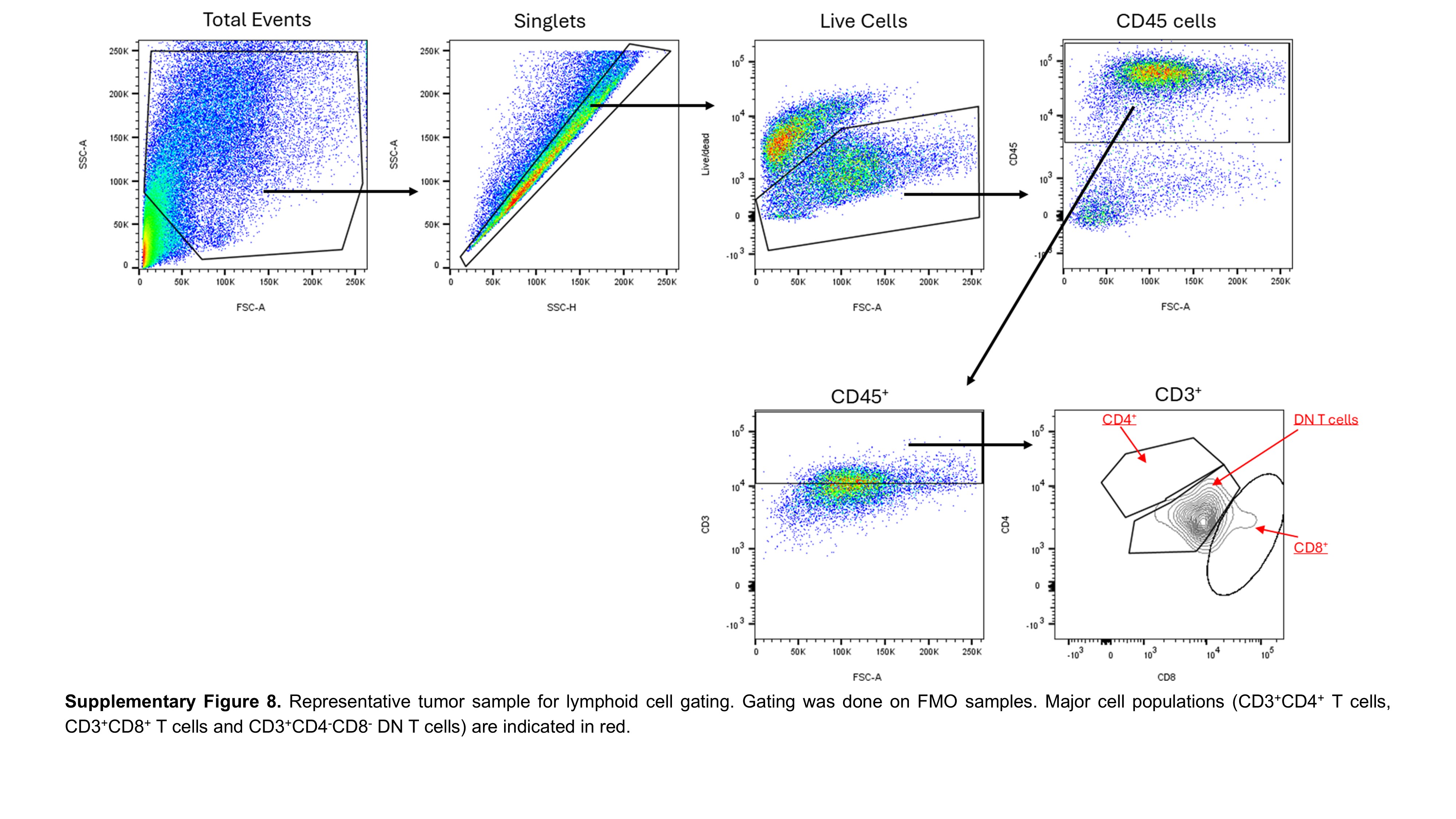

Supplement: Supplementary Figure 8 — Representative tumor sample for lymphoid cell gating. Gating was done on FMO samples. Major cell populations (CD3+CD4+ T cells, CD3+CD8+ T cells and CD3+CD4-CD8- DN T cells) are indicated in red. [file Image8.jpeg]
